# A bioinformatic analysis of the role of TP53 status on the infiltration of CD8^+^ T cells into the tumor microenvironment

**DOI:** 10.1590/1414-431X2023e12970

**Published:** 2023-10-20

**Authors:** A.A. El-Arabey, H.E. Abdel-Hamied, M.E. Awadalla, B. Alosaimi, T.N. Almanaa, S.T. Al-Shouli, Y.A. Modafer, H.W. Alhamdi, M. Abdalla

**Affiliations:** 1 Department of Pharmacology and Toxicology, Faculty of Pharmacy, Al-Azhar University, Cairo, Egypt; 2 Department of General Pathology, Faculty of Medicine for Girls, Al-Azhar University, Cairo, Egypt; 3 Research Center, King Fahad Medical City, Riyadh Second Health Cluster, Riyadh, Saudi Arabia; 4 Department of Botany and Microbiology, College of Science, King Saud University, Riyadh, Saudi Arabia; 5 Immunology Unit, Department of Pathology, College of Medicine, King Saud University, Riyadh, Saudi Arabia; 6 Department of Biology, Faculty of Science, Jazan University, Jazan, Saudi Arabia; 7 Department of Biology, College of Sciences, King Khalid University, Abha, Saudi Arabia; 8 Pediatric Research Institute, Children's Hospital Affiliated to Shandong University, Jinan, Shandong, China

**Keywords:** CD8^+^ T cells, TP53 status, Infiltration, Head and neck cancer, Uterine corpus endometrial carcinoma, Lung adenocarcinoma

## Abstract

CD8^+^ T cells play basic roles in the immune system in a tumor microenvironment (TME) to fight cancer. Several reports have suggested signs of the involvement of tumor protein p53 (TP53) in a complex immune system network. Moreover, our previous research indicated that TP53 orchestrates the polarization and infiltration of macrophages into the TME. In the present study, the clinical function of TP53 status (wild/mutant) in CD8^+^ T cell infiltration was assessed using more than 10,000 The Cancer Genome Atlas (TCGA) samples from 30 cancer types through Tumor Immune Estimation (TIMER). Our investigation revealed that CD8^+^ T cell infiltration was higher in head and neck squamous cell carcinoma (HNSC) and uterine corpus endometrial carcinoma (UCEC) patients with wild-type TP53 than in those with mutant TP53. Wild-type TP53 conferred a good prognosis for HNSC and UCEC (P<0.05). In contrast, CD8^+^ T cell infiltration in lung adenocarcinoma (LUAD) patients with wild-type TP53 was much lower than in those with mutant TP53. Notably, clinical outcomes for LUAD with wild-type TP53 were poor (P<0.05). This study was the first to provide insights into the novel association of TP53 with CD8^+^ T cells infiltration in the TME in patients with HNSC, LUAD, and UCEC. Therefore, TP53 status acts as a prognostic marker, and this can be used as a basis to further study the effect of targeting TP53 in these patients. Furthermore, our study found that TP53 status was a reliable predictive factor and therapeutic target in patients with HNSC and UCEC.

## Introduction

A tumor microenvironment (TME) is an attractive complex cellular environment with an extracellular matrix that encapsulates tumors with immune cells. The immune system includes numerous immune cells, such as T and B lymphocytes, natural killer cells, blood vessels, pericytes, stroma, monocytes/macrophages, adipocytes, and neutrophils. The characteristic of most types of malignancy is immune cell infiltration (1-3). Emerging advanced research has confirmed that immunity of CD8^+^ T cells is an appealing path for cancer immunotherapy. CD8^+^ T cells have a cytotoxic effect on cancer following detection by the immune system. Substantially, T-cell receptor complexes (TCRs) and linked networks become clustered at the contact area between tumor cells and T cells to form an immune synapse (IS) (4). Indeed, active CD8^+^ T cells can kill malignant cells through three scenarios: 1) formation of antitumor cytokines; 2) production of cytotoxic granules with IS, such as perforin, and granzymes, which are also found in nature killer (NK) cells; and 3) destruction of malicious cells via Fas/FasL interactions. Moreover, CD8^+^ T cells can induce an excessive immune response that drives immune-mediated damage (5). CD8^+^ T cell infiltration is the main determinant of the therapeutic effect of immunotherapy on cancers (6). Therefore, tumors can enhance numerous direct and indirect defense mechanisms to abrogate the infiltration capacity and function of CD8^+^ T cells (7). Various complex interactions govern the relationship between tumor infiltration and rejection of CD8^+^ T cells that are still poorly understood (8). As such, the immune cytotoxic effect of CD8^+^ T cells can be abrogated by tumors (9,10).

Tumor protein p53 (TP53) plays a crucial role in numerous cell cycle phases, apoptosis pathways, and genomic stability. The TP53 field of research emerged from the intersection of cancer virus research with immunological methods. It has tracked the progress of cancer research over the last four decades. Cancer treatments are increasingly relying on immunotherapy, and there are various indications that the TP53 protein is crucial in both innate immune system modulation and as an antigen in adaptive immune responses. The TP53 gene and protein are innate immune system components that play essential roles in cancer, ageing, and the recognition of repetitive DNA and RNAs. In cancer, the mutated TP53 protein causes a B-cell antibody response as well as a CD-8 killer T-cell response. The area of research on p53 immune response will be expanded in the future to include cancer immunotherapy, inflammatory reactions, as well as the regulation of epigenetic stability and tissue regeneration (11). Significantly, our recent analysis of The Cancer Genome Atlas (TCGA) demonstrated that TP53 is an attractive target for patients with ovarian serous cystadenocarcinoma (OV) and stomach adenocarcinoma (STAD) through its action as a master regulator of macrophage polarization and infiltration (1).

For several decades, mice have been considered at the core of *in vivo* immunological testing and human biological mirrors because they possess the same protection of immune system components. Although prior to conservation, there are important differences in the sequence of immune system activity and activation between mice and humans (12,13). Present and future experiments should be performed to take advantage of technological advances in manipulating human TCGA data to decipher the complexity and heterogeneity of immune cells (8). Hence, this work was proposed to examine the prospective unknown role of TP53 in the infiltration of CD8+ T cells in different cancer forms by using the clinical data of TCGA.

## Material and Methods

Tumor Immune Estimation (TIMER; http://timer.cistrome.org) is a comprehensive bioinformatics tool to analyze the infiltration of immune cells among different types of cancers. The abundance of CD8^+^ T cells is examined via the TIMER algorithm to access the clinical impact and characteristic genomics (1).

More than 10,000 TCGA samples were examined through TIMER as a bioinformatic method to study the effect of TP53 status on the infiltration rate of CD8^+^ T cells in 30 different cancer types; a comprehensive web server was used to analyze the abundance of different immune cells (14,15). A mutation module was utilized to compare immune CD8^+^ T infiltration levels with and without the involvement of TP53 mutation. Next, box plots of CD8^+^ T cells were drawn to compare the distributions of immune infiltration levels under the TP53 mutation status, and statistical significance was examined via a two-sided Wilcoxon rank-sum test. Kaplan-Meier plots of immune infiltrates and genes were developed to visualize survival differences. A user-defined slider was used to divide the threshold into low and high levels. In each plot, P of the log-rank test for comparing the survival curves of two groups was shown. The TP53 status in various cancers was determined using the TCGA database via TIMER. The somatic copy number alterations (SCNA) module compares tumor infiltration levels between tumors with different somatic copy number alterations for a given gene. GISTIC 2.0 defines SCNAs as deep deletion, arm-level deletion, diploid/normal, arm-level gain, and high amplification. The distributions of each immune subset at each copy number status in selected cancer types are depicted using box plots. A two-sided Wilcoxon rank-sum test compares the infiltration level for each SCNA category to the normal (1).

The prognostic importance of TP53 expression in head and neck squamous cell carcinoma (HNSC), lung adenocarcinoma (LUAD), and uterine corpus endometrial carcinoma (UCEC) was analyzed using the Kaplan-Meier plotter database. The Kaplan-Meier plotter can assess the relationship between the expression of all genes and overall survival in a variety of tumor samples. TCGA is among the databases' sources. The primary goal of the tool is to discover and validate survival biomarkers using meta-analysis. Patient samples were divided into two categories based on different quantile expression levels of potential biomarkers. A Kaplan-Meier survival plot was used to compare two patient cohorts. Hazard ratios with 95% confidence intervals and log-rank P values were estimated (1).

**Figure 1:**
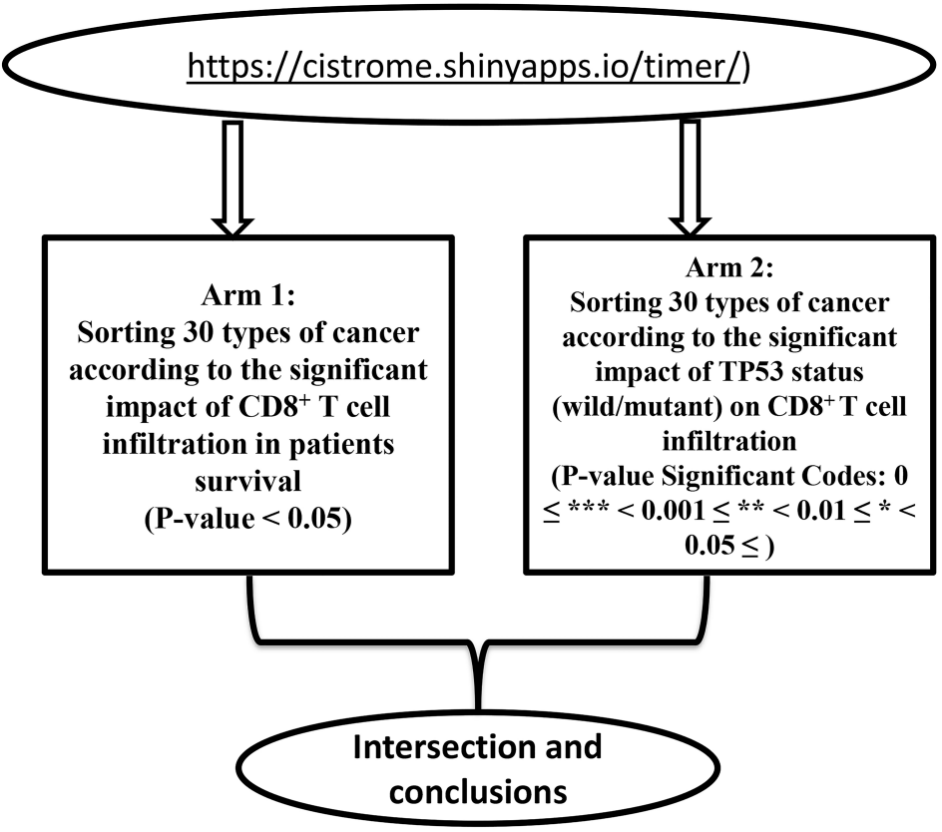
Schematic of the method used in the current study.

Furthermore, we used cBioportal (https://www.cbioportal.org) as a platform to compare the overall survival of patients with wild-type TP53 against mutant TP53. Our analysis was divided into two arms: Arm 1 (sorting 30 forms of cancer in terms of the clinical effect of CD8^+^ T cell infiltration on patient survival) and Arm 2 (sorting 30 forms of cancer in terms of the main influence of TP53 status (wild/mutant) on CD8^+^ T cell infiltration) (Figure 1). The intersection between the two arms of the current study was created by utilizing the Venny tool to obtain the cross-targets (16). In addition, we utilized an integrated repository portal for tumor-immune system interactions (TISIDB) as a web portal for tumor and immune system interaction, integrating multiple heterogeneous data. TISIDB was used to investigate the connections between tumor-infiltrating lymphocyte abundance and TP53 mutation. Using gene set variation analysis based on gene expression profiles, the relative abundance of CD8^+^ T cells was calculated for each cancer type (1,4).

## Results

### TP53 status orchestrated the level of CD8^+^ T cell infiltration in HNSC, KICH, LUAD, STAD, and UCEC

**Figure 2:**
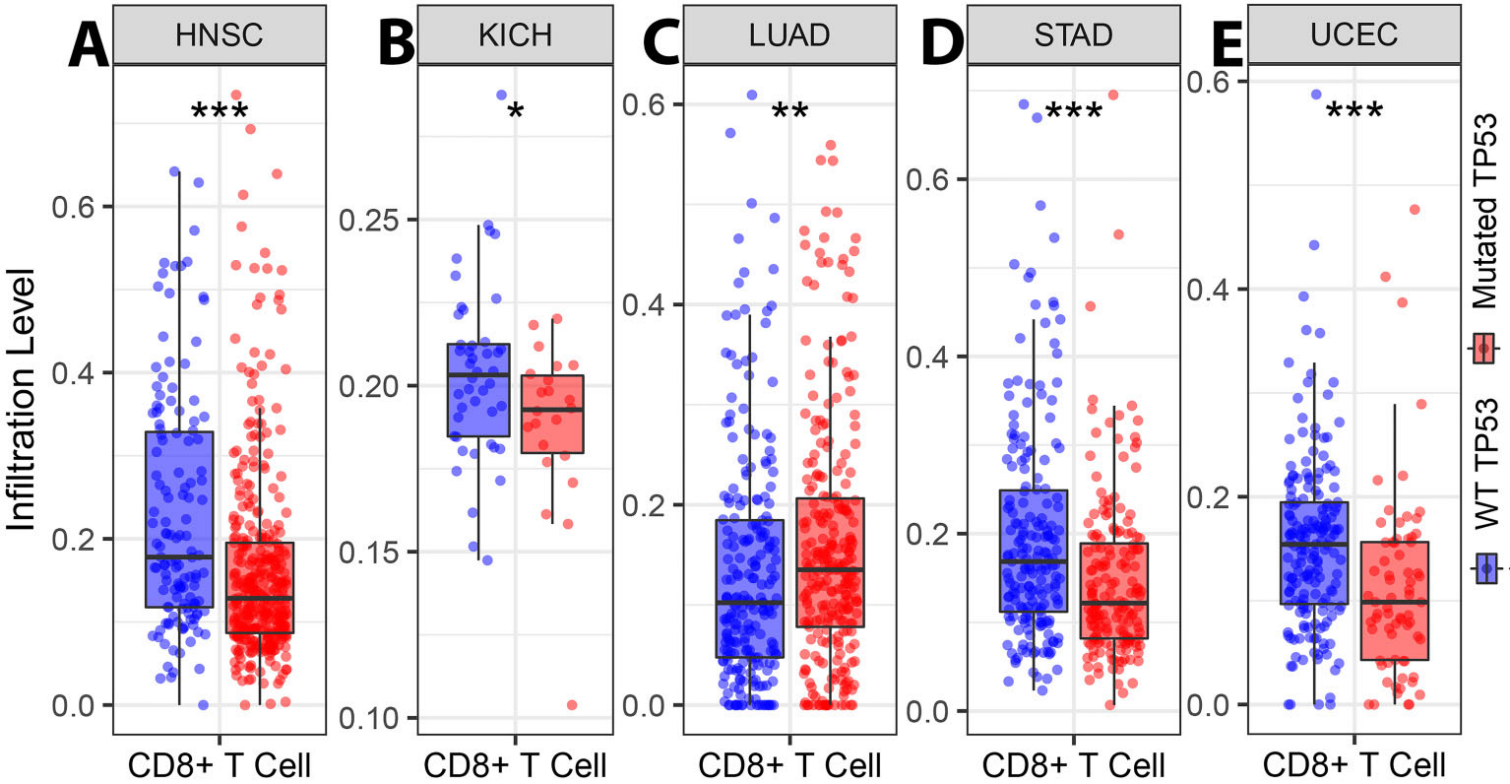
TP53 status and CD8^+^ T cell infiltration. CD8^+^ T cell infiltration levels of wild-type and mutant TP53 in head and neck squamous cell carcinoma (HNSC; **A**) (P≤0.001), kidney chromophobe (KICH; **B**) (P≤0.05), lung adenocarcinoma (LUAD; **C**) (P≤0.01), stomach adenocarcinoma (STAD; **D**) (P≤0.001), and uterine corpus endometrial carcinoma (UCEC; **E**) (P≤0.001). Data are reported as median and interquartile range (Wilcoxon test).

**Figure 3:**
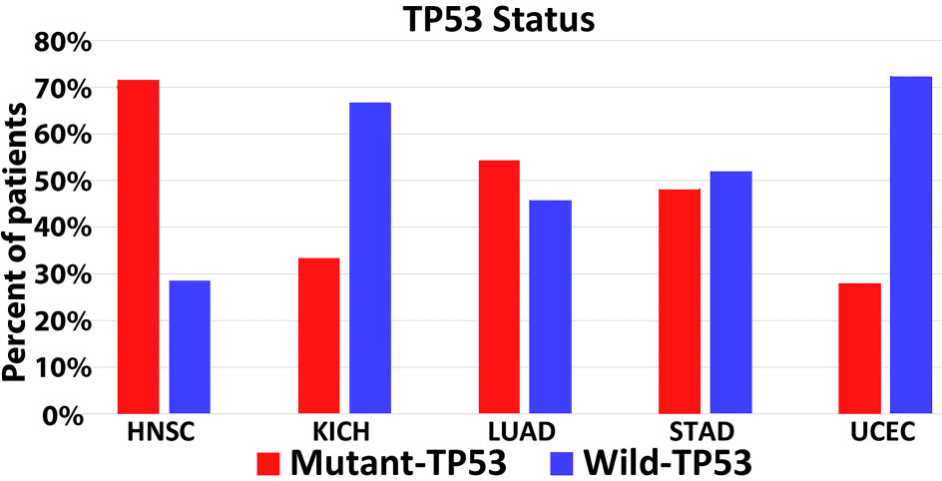
TP53 status of head and neck squamous cell carcinoma (HNSC), kidney chromophobe (KICH), lung adenocarcinoma (LUAD), stomach adenocarcinoma (STAD), and uterine corpus endometrial carcinoma (UCEC) via Tumor Immune Estimation (TIMER).

Through TIMER, our study showed that TP53 status significantly affected the level of CD8^+^ T cell infiltration in 5 out of 30 cancer types, including HNSC, kidney chromophobe (KICH), LUAD, STAD, and UCEC (Figure 2; Supplementary Figures S1-S3). Notably, our data illustrated that the CD8^+^ T cell infiltration levels of patients with HNSC, KICH, STAD, and UCEC and wild-type TP53 were significantly higher than those with mutant TP53 (P<0.05; Figure 2A, B, D, and E). Conversely, the infiltration of CD8^+^ T cells of patients with mutant TP53 and LUAD was significantly higher than those with wild-type TP53 (P<0.05; Figure 2C). In addition, the ratio of wild-type and mutant TP53 was established in these five forms of cancer. Our analysis showed that the mutation percentages of TP53 in HNSC, KICH, LUAD, STAD, and UCEC were 71.5, 33.3, 54.3, 48.1, and 27.8%, respectively (Figure 3).

### TP53 status had a significant clinical effect on the infiltration rate of CD8^+^ T cells and survival of patients with HNSC, LUAD, and UCEC

**Figure 4:**
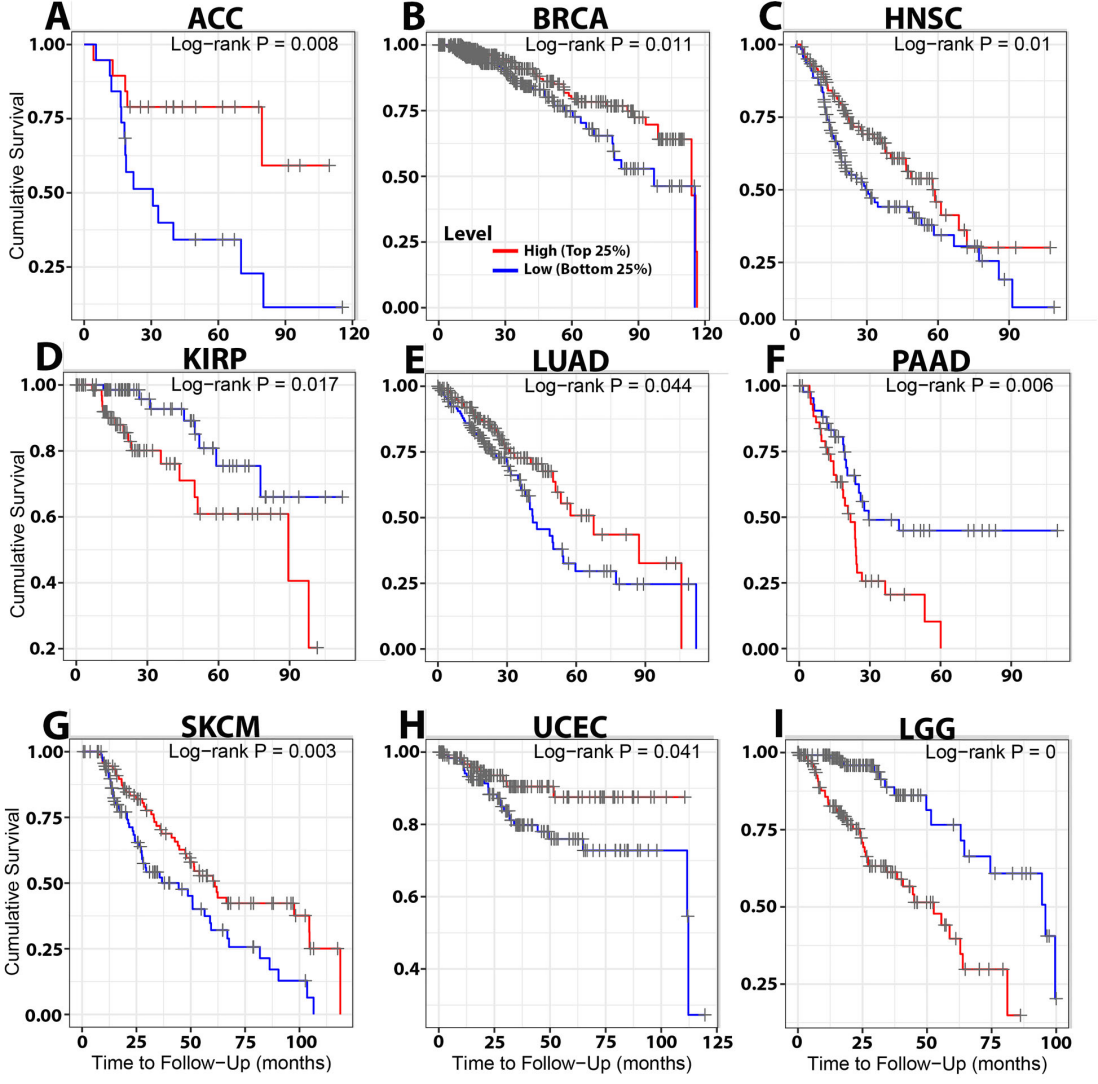
The Cancer Genome Atlas (TCGA) survival analysis via Tumor Immune Estimation (TIMER) showed that high infiltration of CD8^+^ T cells was associated with good prognosis in patients with ACC (log-rank P=0.008; **A**), BRCA (log-rank P=0.011; **B**), HNSC (log-rank P=0.01; **C**), LUAD (log-rank P=0.044; **E**), SKCM (log-rank P=0.0083; **G**), and UCEC (log-rank P=0.041; **H**). In contrast, low infiltration of CD8^+^ T cells was related to good prognosis in patients with KIRP (log-rank P=0.017; **D**), PAAD (log-rank P=0.006; **F**), and LGG (log-rank P=0; **I**). For explanation of abbreviations, see Table 1.

**Figure 5:**
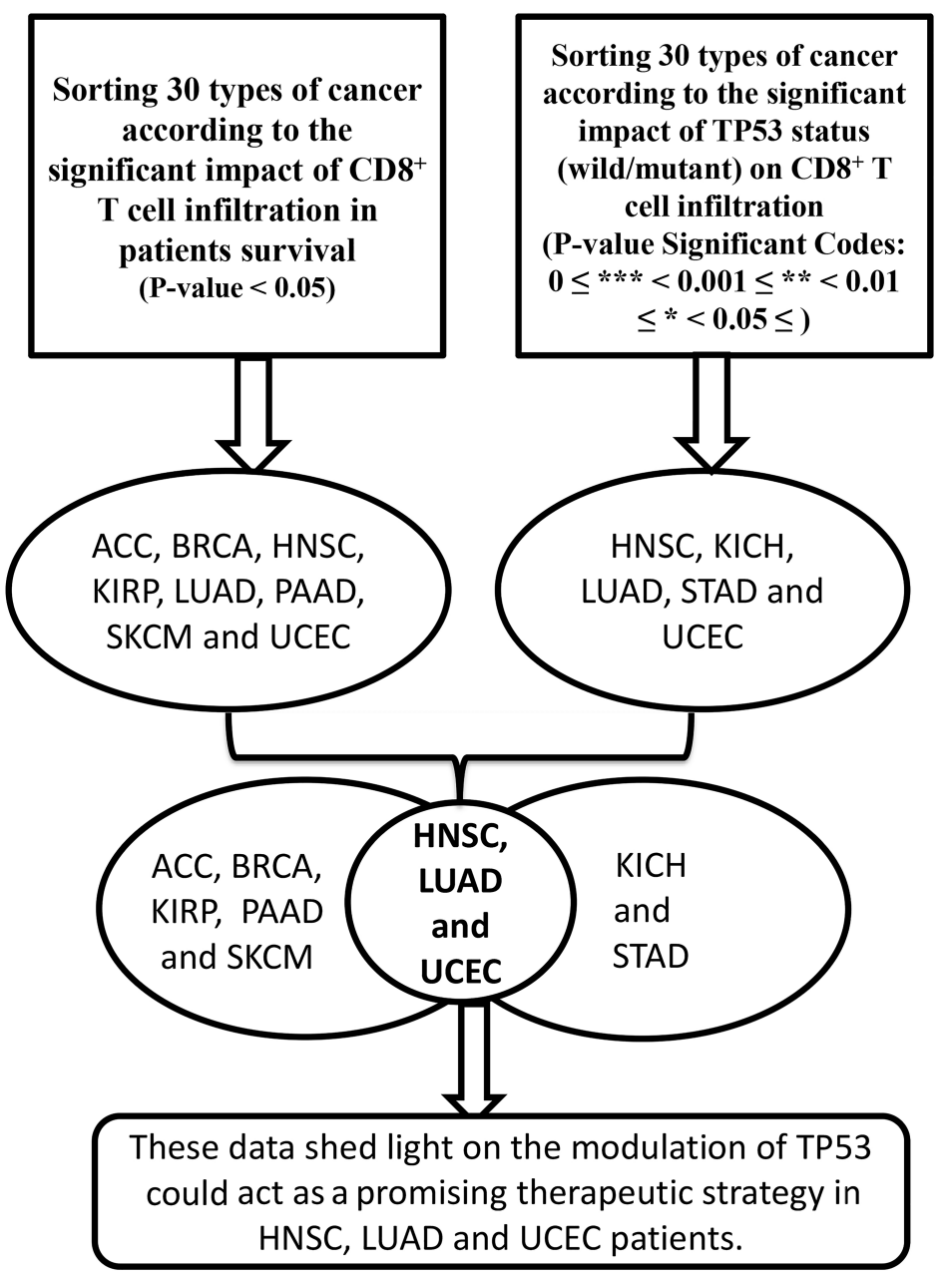
Schematic showing the significant results of the two arms of our study and the overlapping result between both arms with brief conclusions. For explanation of abbreviations, see Table 1.

TIMER was utilized to examine the effect of the infiltration rate of CD8^+^ T cells on the patients’ survival of 30 cancer forms. In clinical aspects, our results showed that a high infiltration rate of CD8^+^ T cells was substantially associated with positive clinical outcomes in ACC, BRCA, HNSC, LUAD, SKCM, UCEC, and LGG (P<0.05; Table 1; Figure 4A, B, C, E, G, H, and I) but not significant in other cancers (Supplementary Table S1). Our analysis confirmed that the infiltration rate of CD8^+^ T cells of patients with kidney renal papillary cell carcinoma (KIRP) and pancreatic adenocarcinoma (PAAD) was higher, and their survival was considerably poorer than that of patients with a lower CD8^+^ T cell infiltration rate (P<0.05; Figure 4D and F). In this regard, an intersection was made between the effects of the two arms of our proposal using Venny to capture the cross-target of cancers. The outcome of this intersection showed that the infiltration rate of CD8^+^ T cells and the clinical effect on the survival of patients with HNSC, LUAD, and UCEC were orchestrated by TP53 status (Figure 5).

**Table 1:** CD8^+^ T cell impact on survival for 30 cancers via Tumor Immune Estimation (TIMER).

Type of Cancer	Survival analysis
Adrenocortical Carcinoma (ACC)	**0.008**
Bladder Urothelial Carcinoma (BLCA)	0.113
Breast Invasive Carcinoma (BRCA)	**0.011**
Cholangiocarcinoma (CHOL)	0.331
Colon Adenocarcinoma (COAD0	0.104
Diffuse Large B-Cell Lymphoma (DLBC)	0.571
Esophageal Carcinoma (ESCA)	0.923
Glioblastoma Multiform (GBM)	0.542
Head and Neck Cancer (HNSC)	**0.010**
Kidney Chromophobe ((KICH)	0.91
Kidney Renal Clear Cell Carcinoma (KIRC)	0.094
Kidney Renal Papillary Cell Carcinoma (KIRP)	**0.017**
Lower Grade Glioma (LGG)	**0.000**
Liver Hepatocellular Carcinoma (LIHC)	0.391
Lung Adenocarcinoma (LUAD)	**0.044**
Lung Squamous Cell Carcinoma (LUSC)	0.435
Mesothelioma (MESO)	0.070
Ovarian Serous Cystadenocarcinoma (OV)	0.400
Pancreatic Adenocarcinoma (PAAD)	**0.006**
Pheochromocytoma Paraganglioma (PCPG)	0.285
Prostate Carcinoma (PRAD)	0.548
Rectum Adenocarcinoma (READ)	0.593
Sarcoma (SARC)	0.169
Stomach Adenocarcinoma (STAD)	0.837
Skin Cutaneous Carcinoma (SKCM)	0.003
Thymoma (THYM)	0.360
Testicular Germ Cell Tumors (TGCT)	0.973
Thyroid Carcinoma (THCA)	0.270
Uterine Corpus Endometrial Carcinoma (UCEC)	**0.041**

Bold type indicates statistical significance (log-rank P value).

**Figure 6:**
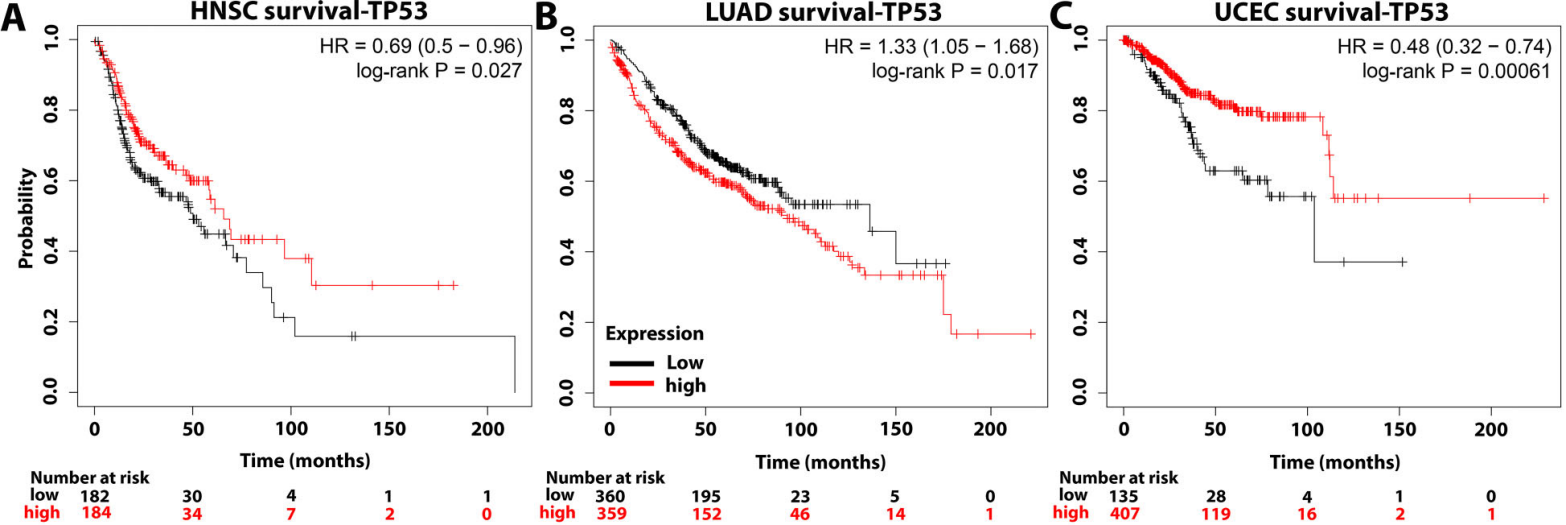
Survival analysis via a Kaplan-Meier plotter for TP53 expression in head and neck squamous cell carcinoma (HNSC, **A**), lung adenocarcinoma (LUAD, **B**), and uterine corpus endometrial carcinoma (UCEC, **C**). Log-rank P=0.027, 0.017, and 0.00061, respectively.

**Figure 7:**
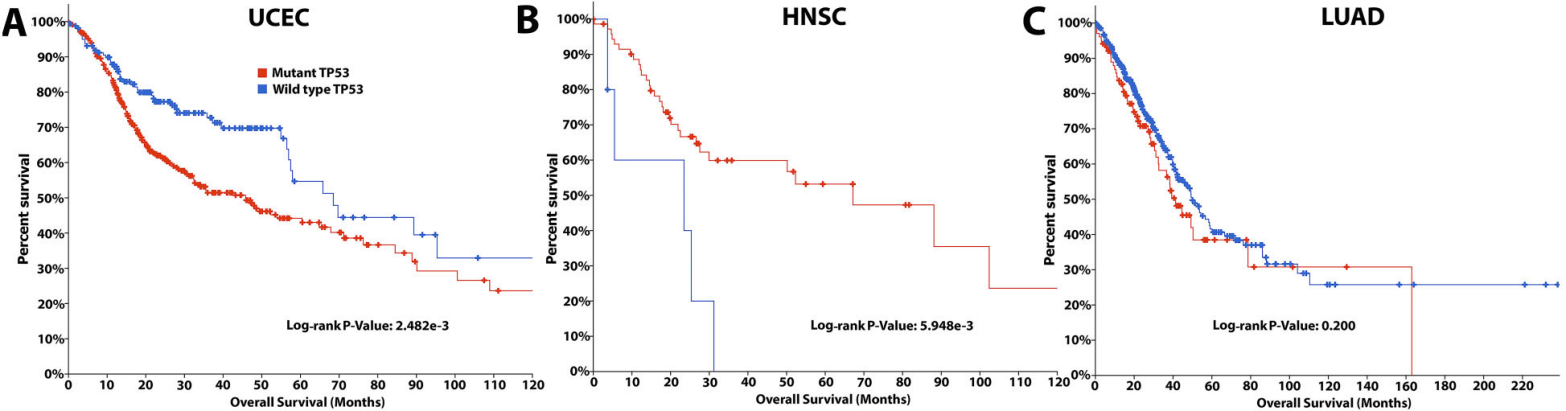
The Cancer Genome Atlas survival analysis via cBioportal for mutant/wild-type TP53 in patients with uterine corpus endometrial carcinoma (UCEC, **A**), head and neck squamous cell carcinoma (HNSC, **B**), and lung adenocarcinoma (LUAD, **C**).

**Figure 8:**
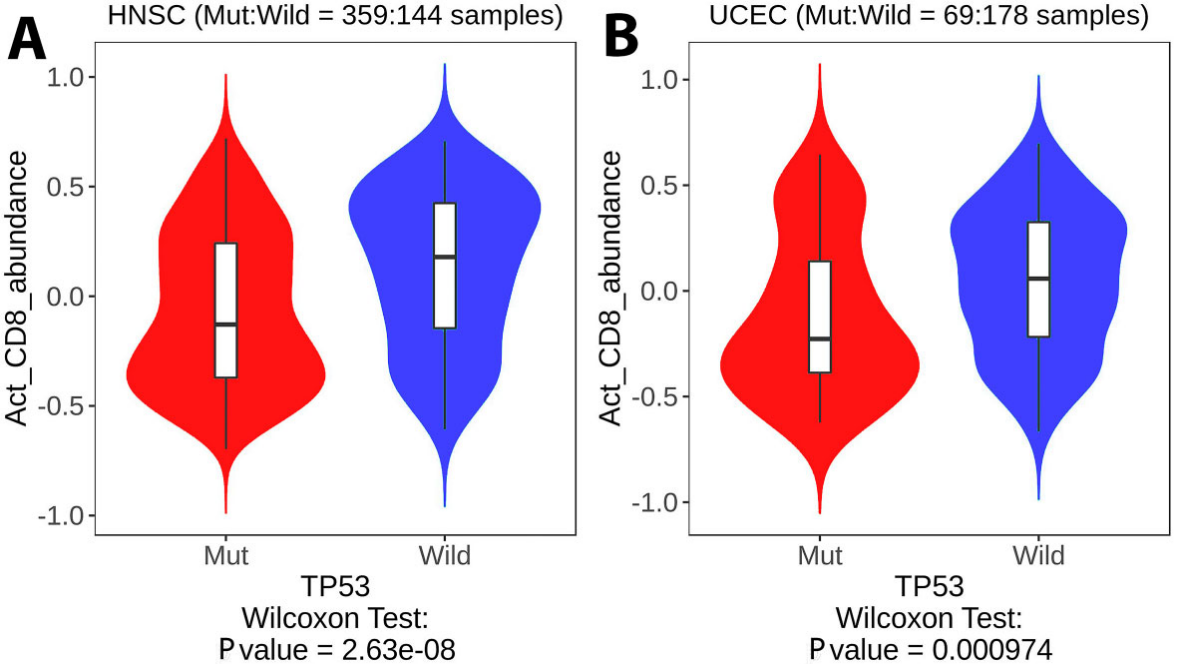
The impact of TP53 status (wild/mutant) on the number of activated CD8^+^ T lymphocytes in patients with head and neck squamous cell carcinoma (HNSC) and uterine corpus endometrial carcinoma (UCEC). Wild-type TP53 is significantly related to the quantity of activated CD8^+^ T lymphocytes in HNSC (**A**) and UCEC (**B**) patients compared to mutant (Mut) TP53.

The TCGA data of the Kaplan-Meier plotter were used to investigate the clinical impact of TP53 expression on patients with HNSC, LUAD, and UCEC and validate our previous results. Notably, for patients with HNSC (P<0.05; Figure 6A) and UCEC (P<0.05; Figure 6C), high TP53 expression was predictive of a positive prognosis. By comparison, high TP53 expression predicted a poor prognosis of patients with LUAD (P<0.05; Figure 6B). Furthermore, our study on TCGA data via the cBioportal platform revealed that the overall survival of patients with UCEC and mutant TP53 was shorter than that of patients with wild-type TP53 (Figure 7A). The average survival of patients with HNSC and mutant TP53 was longer than that of patients with wild-type TP53 (Figure 7B). However, comparing wild-type TP53 to mutant TP53 in patients with LUAD revealed no significant variations (Figure 7C). Following that, we used the TISIDB database to investigate the influence of TP53 status on the number of activated CD8^+^ T lymphocytes in HNSC and UCEC patients. Notably, compared to mutant TP53, wild-type TP53 was significantly associated with abundance of activated CD8^+^ T lymphocytes in HNSC and UCEC patients (Figure 8A and B).

Next, we compared the extent of CD8^+^ T cell infiltration in patients with HNSC, LUAD, and UCEC with that of patients through the numerous SCNA modules (GISTIC 2.0) for TP53 using the two-sided Wilcoxon rank sum test. In patients with HNSC, the infiltration of CD8^+^ T cells was significantly lower in the categories of arm-level gain (P<0.001) and arm-level deletion (P<0.01) than the normal levels (Supplementary Figure S4A). Similarly, our findings on patients with UCEC indicated that the infiltration rate of CD8^+^ T cells was considerably lower in arm-level gain (P<0.001) and arm-level deletion (P<0.001) relative to standard levels (Supplementary Figure S4B). By contrast, our analysis revealed no vital effect on the different forms of SCNA modules for patients with LUAD (Supplementary Figure S4C). CD8^+^ T cell infiltration level of wild-type TP53 was higher than that of mutant TP53 in STAD (P≤0.001) and in UCEC (P≤0.001).

## Discussion

The transformed extracellular matrix of the TME comprises multiple types of immune cells to drive immune evasion and tumor formation (17). The extracellular matrix has a crucial role in the production of noncellular components, such as cytokines, chemokines, and other soluble factors. Subsequently, the TME is a network of active interactions between immune cells and tumor cells (18). The role of T cells depends on their association and interaction with tumor cells or other TME cells (19). Many key factors are strongly dependent on the antitumor effect of CD8^+^ T cells, such as the differentiation of CD8^+^ T cells and the infiltration rate of CD8^+^ T cells into the TME (20). TP53 has a crucial role in regulating cellular stress, DNA damage repair, apoptosis, cell cycle arrest, and senescence. Moreover, several studies have shown that TP53 prevents autoimmune inflammation and intrinsic immune reactions. TP53 has been shown to be involved in the infiltration rate (1) and physiological breakdown of macrophage polarization via the TP53/MDM2 axis (1,21). Wild-type TP53 tumor cells rapidly undergo CD8^+^ T cell-induced apoptosis, whereas tumor cells with P53 mutation are immune to CD8^+^ T cell-mediated apoptosis (22). However, the main role of TP53 in the infiltration rate of CD8^+^ T cells remains unknown.

In the current study, we found for the first time that the status of TP53 significantly regulated the infiltration of CD8^+^ T cells in HNSC, KICH, LUAD, STAD, and UCEC patients. Furthermore, the high infiltration rate of CD8^+^ T cells in patients with ACC, BRCA, HNSC, KIRP, LUAD, PAAD, SKCM, and UCEC was associated with a positive clinical response. Collectively, the intersection of the two arms of our research provided insights into the clinical therapeutic function of TP53 as a basis for determining the infiltration rate of CD8^+^ T cells in patients with HNSC, LUAD, and UCEC (Figure 5).

The significant antitumor effect of the infiltration rate of CD8^+^ T cells in patients with HNSC was identified in a recent pioneering study by Saloura et al. (23). The infiltration rate of CD8^+^ T cells in patients with LUAD was higher than in patients with squamous cell carcinoma (24). In patients with UCEC, CD8^+^ T cells have been shown to be correlated with patient survival (25). The infiltration rate of CD8+ T cells in patients with HNSC and UCEC and mutant TP53 (71.5 and 27.8%, respectively) was lower than that in patients with wild-type TP53 (28.5 and 72.2%, respectively). Previous studies showed that TP53 participates in CD8+ T cells homeostasis (26). In the absence of IFNγ treatment, TP53 stimulation increases the major histocompatibility complex I (MHCI)-antigen presentation in cancer cells, which harbor wild-type TP53 through the activation of the endoplasmic reticulum aminopeptidase ERAP1 (27). The important mechanism used by CD8^+^ T cells to induce their antitumor effect is the expression of Fas ligand (FasL) in the SI within the interaction of tumor with CD8^+^ T cells (28). Nonetheless, several reports have demonstrated that CD8^+^ T cells mediate cancer cell apoptosis through the activation of Fas/APO-1 after MHCI recognition (22).

**Figure 9:**
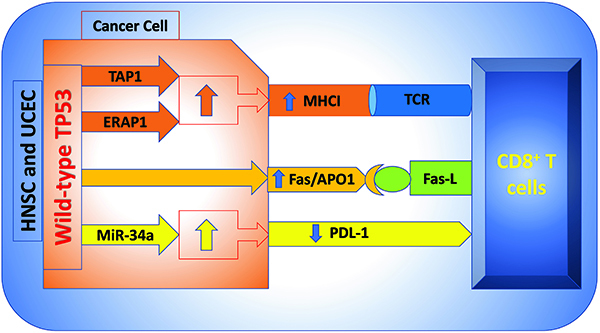
Schematic showing the possible regulatory pathway of CD8^+^ T cell infiltration in patients with head and neck squamous cell carcinoma (HNSC) and uterine corpus endometrial carcinoma (UCEC) through wild-type TP53. TAP1: transporter associated with antigen processing 1 (TAP1); ERAP1: endoplasmic reticulum aminopeptidase; ERAP1; MiR-34a: micro-RNA-34a.

Furthermore, wild-type TP53 regulates the PD-L1 expression by binding to the promoter region of miR-34a. Consequently, the decreased expression of this immune checkpoint increases the probability of CD8^+^ T cell-mediated tumor cell apoptosis (29). In contrast, LUAD and non-small cell lung cancer have downregulated miR-34a levels. Therefore, the interaction between TP53 and miR-34a appears essential for controlling CD8^+^ T cells infiltration and is responsible for the opposite phenotype of CD8^+^ T cells in cancers (30). Significantly, TP53 induces the transporter associated with antigen processing 1 (TAP1) to improve the peptide transport of MHCI and the surface expression of MHC-peptide complexes. TP53 contributes to IFNγ to stimulate the MHCI pathway (31). Similarly, our results showed that the infiltration rate of CD8^+^ T cells in patients with HNSC and UCEC and with mutant TP53 was lower than that in patients with wild-type TP53. It is well known that cross-priming, the process by which dendritic cells activate CD8^+^ T cells by presenting exogenous antigens to them, is essential in generating anti-tumor CD8^+^ T cell immunity (31). Tumor-driven microenvironments offer the required circumstances for controlling infiltrating CD8^+^ T cells in favor of tumor survival, such as weakening CD8^+^ T cell activation, directing tumor cells to impede immunological attack, and recruiting other cells to remodel the immune milieu (31). In this sense, our results showed the effect of TP53 status on the number of activated CD8^+^ T lymphocytes in patients with HNSC and UCEC. Wild-type TP53 was significantly associated with the abundance of activated CD8^+^ T lymphocytes in HNSC and UCEC patients compared to mutant TP53. Hence, these data provided insights into the clinical impact of TP53 as an immunomodulator and promising therapeutic strategy in patients with HNSC and UCEC to orchestrate the infiltration rate of CD8^+^ T cells (Figure 9). Therapeutic cancer vaccines stimulate the immune system to attack existing cancer. Over twenty clinical trials have used vaccines to target or boost TP53 in malignant disease (32). Hence, vaccination with a TP53 peptide to boost immune responses to HNSC and UCEC patients is highly promising.

On the other hand, even though the infiltration rate of CD8^+^ T cells in LUAD patients with wild-type TP53 was much lower than in those with mutant TP53, our survival study revealed that TP53 expression, rather than TP53 status, had an influence on the survival of LUAD patients. Our findings are consistent with a previous study that concluded that the TP53 deficient gene profile, rather than just TP53 mutant status, is a strong predictor of overall survival and medication sensitivity in various cancer types and therapies such as LUAD (31). Furthermore, several studies have reported that TP53 mutation is significantly associated with immunotherapy response in LUAD patients but is not an ideal independent prognostic predictor of it (33). Therefore, it seems that other special factors such as tumor mutation burden are linked with immune infiltrate in LUAD-mutant TP53 patients (33,34).

### Conclusions

Our bioinformatic analyses showed novel evidence that TP53 status is strongly associated with the infiltration levels of CD8^+^ T cells into the TME of HNSC, LUAD, and UCEC patients. However, TP53 status of HNSC and UCEC malignancies was highly associated with patient survival via several mechanisms. In this regard, wild-type TP53 significantly correlated to abundance of activated CD8^+^ T lymphocytes in HNSC and UCEC patients compared to mutant TP53. As a result, our findings shed light on an appealing strategy for the treatment of HNSC and UCEC patients.

## Supplementary Material

Click here to view [pdf].
